# Dual polarization of human alveolar macrophages progressively increases with smoking and COPD severity

**DOI:** 10.1186/s12931-017-0522-0

**Published:** 2017-02-23

**Authors:** Erica Bazzan, Graziella Turato, Mariaenrica Tinè, Claudia M. Radu, Elisabetta Balestro, Chiara Rigobello, Davide Biondini, Marco Schiavon, Francesca Lunardi, Simonetta Baraldo, Federico Rea, Paolo Simioni, Fiorella Calabrese, Marina Saetta, Manuel G. Cosio

**Affiliations:** 1 Department of Cardiac, Thoracic, and Vascular Sciences, University of Padova, Padova, Italy; 2 Department of Medicine, University of Padova, Padova, Italy; 3 Respiratory Division, Meakins-Christie Laboratories, McGill University, Montreal, Canada

**Keywords:** COPD, Cigarette smoking, Macrophages, M1, M2

## Abstract

**Background:**

It is known that tissue macrophages derive not only from blood monocytes but also from yolk sac or fetal liver, and the tissue of residence guides their function. When isolated, they lose tissue specific signatures, hence studies of human macrophages should be ideally done directly in the tissue. The aim of this study was to investigate directly in human lung tissue the polarization of alveolar macrophage (AM), classic (M1) or alternative (M2), in health and disease, using COPD as a model.

**Methods:**

Surgical lungs from 53 subjects were studied: 36 smokers whose FEV_1_ varied from normal to severe COPD, 11 non-smokers and 6 normal donors. iNOS and CD206 immunohistochemistry was used to quantify the percentage of AM polarized as M1 or M2 in lung sections.

**Results and Discussion:**

The percentage of M1 and M2 increased progressively with smoking and COPD severity, from 26% to 84% for M1 and from 7% to 78% for M2. In donors 74% of AM were negative for M1 and 93% for M2. Confocal microscopy showed co-localization of M1 and M2 in the same AM in severe COPD.

**Conclusion:**

In normal lungs alveolar macrophages were mostly non-polarized. With smoking and COPD severity, M1 and M2 polarization increased significantly and so did the co-expression of M1 and M2 in the same alveolar macrophage.

**Electronic supplementary material:**

The online version of this article (doi:10.1186/s12931-017-0522-0) contains supplementary material, which is available to authorized users.

## Background

The concept of macrophages (big eaters) as professional phagocytes playing a critical role in the degradation of endogenous and foreign materials has evolved greatly. It is now evident that macrophages, which are present in almost all tissues, coordinate immunological, metabolic and developmental functions contributing to the maintenance of homeostasis. Furthermore, when the host is challenged by infection or injury, macrophages play a critical role in the coordination of host defense and eventually tissue repair [1, 2].

Upon encountering pathogens or danger signals, macrophages express a strong pro-inflammatory profile including cytokines and reactive oxygen and nitrogen species. This pro-inflammatory phenotype is recognized as the “classically activated” or M1 phenotype and can be produced in vitro in response to inflammatory stimuli like Lipopolysaccharides (LPS) or Interferon (IFN)-γ [3–5]. By contrast, homeostatic signals, as well as Th-2 cytokines in vitro, induce macrophages to adopt phenotypes linked with tissue remodeling and repair; this phenotype is generally recognized as “alternatively activated” or M2 phenotype [3–5]. Although it is clear that the M1/M2 classification might be simplistic [6, 7] since a spectrum of macrophage phenotypes has been observed in *in vitro* experiments [5], the M1/M2 nomenclature [8] is still widely used and is still the basis for the description of macrophage behavior in human diseases as evidenced in recent publications [9–14].

It is now well established that tissue macrophages derive not only from circulating bone marrow-originated monocytes [15] but also from either the yolk sac (brain, liver, heart) or fetal liver (lung, gut) [16], and that these macrophages are maintained in adult organs independently of circulating monocytes [16]. In the steady state, monocytes do not contribute substantially to tissue macrophages with the exception of the gut, the dermis and the heart. It has now become evident that the unique genomic signatures of tissue macrophages are strongly related to the tissue environmental signals and maintained by local cues [17] and that, when isolated and cultured, tissue macrophages rapidly lose their tissue specific signatures [18, 19]. This new knowledge ought to influence the way macrophages are studied, since tissue environment and specific tissue stimuli would dictate macrophage endotype, phenotype and behavior [2, 17]. Based on the new knowledge about macrophage dependence on the tissue of residence, we thought that ideally the investigation of the macrophage polarization during a human disease ought to be done directly in the tissue, the lung in our case, a methodology that does not require cell isolation thus avoiding the possibility of inducing in vitro artefacts.

We thought that lung response to cigarette smoke exposure and the consequent development of COPD, a chronic and progressive inflammatory disease, could be a model that fulfills these characteristics. For these reasons, we decided to study directly in human lung tissue the pattern of alveolar macrophage (AM) polarization, classic (M1) or alternative (M2), and examine how this pattern changes from the normal lung to a progressive inflammatory disease, COPD, in which the trigger is known (cigarette smoking) and the evolution of the disease can be studied functionally and pathologically.

## Methods

### Subject characteristics

Fifty-three lungs from subjects undergoing lung surgery were studied. Eleven were smokers with severe COPD who had lung volume reduction surgery and no lung tumour; 25 were smokers who had surgery for peripheral malignant nodules of which 12 had moderate COPD and 13 normal lung function; 17 were non-smokers of which 11 had surgery for lung tumour (5 malignant and 6 benign) and 6 died of accidental death (donors). Except for donors, pulmonary function tests were performed shortly before surgery, and to define COPD the post-bronchodilator ratio of forced expiratory volume in one second over forced vital capacity (FEV_1_/FVC) <70% was used. None of the patients had a history of exacerbations or pulmonary infections in the month prior to surgery or history of atopy or asthma.

### Immunohistochemical and confocal analysis

Lungs were fixed in 4% formaldehyde and tissue blocks were taken from the subpleural areas of the lung as far as possible from the tumour, and embedded in paraffin [20]. Sections 5 μm thick were cut and processed for immunohistochemical analysis. For the identification of the AM M1 phenotype we used anti-iNOS (inducible isoform nitric oxide synthases) [10, 11, 21–25] and confirmed the results by using anti-HLA-DR (Human Leukocyte Antigen - antigen D Related) [23–27]. CD206 expression was used for the identification of the AM M2 phenotype [10, 12–14, 28–30]. Additionally, in a subgroup of patients, the expression of Tumour Necrosis Factor (TNF)-α, Interleukin (IL)-4 and IL-13 in AM was investigated by immunohistochemical analysis as indexes of M1 (TNF-α) and M2 (IL-4 and IL-13) polarization [5, 10, 31, 32] (Additional file 1).

Positive alveolar macrophages, defined as mononuclear cells with a well-represented cytoplasm, present in the alveolar spaces, were quantified in at least 20 non-consecutive high-power fields inside the alveolar spaces in each subject. Results were expressed as percentage of positive macrophages over the total number of macrophages visualized. The quantification of iNOS^+^ (M1) and CD206^+^ (M2) AM was performed on two consecutive sections. In two cases from each group, confocal microscopy was also performed to study the possible co-expression of iNOS and CD206. Sections were coded and the measurements made without knowledge of clinical and functional data. Details are reported in the Additional file 1.

### Statistical analysis

Group differences were evaluated by analysis of variance (ANOVA) and unpaired Student t test for clinical data, and by Kruskal–Wallis test and Mann–Whitney U test for morphological data. Correlation coefficients were calculated by the Spearman rank method. Details are reported in the Additional file 1.

## Results

Table 1 shows the clinical characteristics in the 5 groups of subjects examined. No demographic differences were observed among the five groups of subjects. The smoking history was similar in the groups of severe COPD, moderate COPD and smokers without COPD. As expected from the selection criteria, subjects with severe and moderate COPD had lower values of FEV_1_ and FEV_1_/FVC as compared to smokers without COPD and non-smokers.

**Table 1:** Clinical Characteristics of the subjects in the study cohort

	Severe COPD	Moderate COPD	Smokers w/o COPD	Non Smokers	Donors
Subjects examined (nM/nF)	9 M/2 F	11 M/1 F	13 M	6 M/5 F	4 M/2 F
Age, yrs	62 ± 9	66 ± 8	63 ± 8	62 ± 14	56 ± 6
Smoking history, pk-yrs	46 ± 28	50 ± 19	44 ± 23	-	-
FEV_1_, % pred	33 ± 9 †‡	68 ± 9‡	100 ± 10	106 ± 17	-
FEV_1_/FVC (%)	36 ± 11 †‡	64 ± 5‡	77 ± 7	79 ± 4	-
PaO_2_, mmHg	65 ± 14 †‡	81 ± 6	87 ± 8	82 ± 4	-
PaCO_2_, mmHg	40 ± 6	41 ± 4	40 ± 11	38 ± 3	-

Values are expressed as mean ± SD

†Significantly different from patients with moderate COPD (*p <* 0.005)

‡Significantly different from smokers without (w/o) COPD and non-smokers (*p <* 0.0001)

**Fig. 1:**
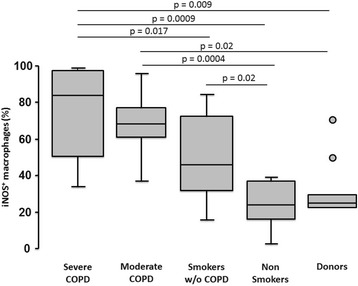
Analyses of M1 polarized alveolar macrophages (iNOS^+^) in the study cohort. The percentage of M1 (iNOS^+^) alveolar macrophages was low in donors and non-smokers, and increased progressively with smoking and COPD severity. Two ventilated subjects in the donors group are identified as outliers (circles). Bottom and top of each box plot, 25^th^ and 75^th^ percentiles; solid line, median; brackets, 10^th^ and 90^th^ percentiles

**Fig. 2:**
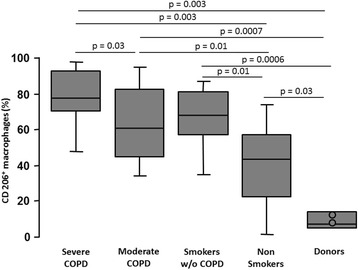
Analyses of M2 polarized alveolar macrophages (CD206^*+*^) in the study cohort. The percentage of M2 (CD206^+^) alveolar macrophages was low in donors, and increased with smoking and COPD severity. Two ventilated subjects in the donors group are identified (circles). Bottom and top of each box plot, 25^th^ and 75^th^ percentiles; solid line, median; brackets, 10^th^ and 90^th^ percentiles

The percentage of iNOS^+^ AM (M1 polarization) increased progressively with smoking and disease severity, from 26% in donors to 84% in severe COPD (Fig. 1). Two of the donors who were ventilated for more than 24 hours, a procedure known to induce lung inflammation [33], showed a twofold increase in the percentage of M1 AM (identified as outliers in the figure) compared with the non-ventilated donors (Fig. 1). The percentage of AM expressing CD206 (M2) also increased with smoking and disease severity, from 7% in donors to 78% in severe COPD (Fig. 2). Interestingly, the percentage of M2 AM was higher in non-smokers compared to donors, possibly because of the presence of lung tumours in non-smokers surgical lungs. Indeed, when non-smokers were divided according to the type of tumour (Additional file 1: Figure S1), CD206 expression was only increased in subjects with malignant tumour but not in those with benign tumour, suggesting that malignancy could influence the expression of M2 in the lung parenchyma. However, it should be noted that patients with severe COPD (Fig. 2), whose lungs were obtained by lung volume reduction surgery and had no lung cancer, had the highest percentage of M2, indicating that most of the increase of M2 is secondary to smoking and COPD severity rather than to the presence of tumour itself.

**Fig. 3:**
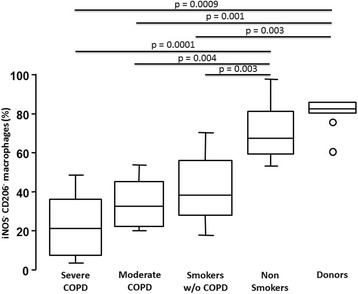
Analyses of non-polarized alveolar macrophages (iNOS^−^ and CD206^−^) in the study cohort. The percentage of non-polarized alveolar macrophages (iNOS^−^CD206^−^) was high in donors and non-smokers and decreased progressively with smoking and COPD severity. Bottom and top of each box plot, 25^th^ and 75^th^ percentiles; solid line, median; brackets, 10^th^ and 90^th^ percentiles

In healthy lungs of donors, more than 80% of AM did not express any polarization marker and this percentage decreased progressively with smoking and disease severity to 20% non polarized AM in severe COPD (Fig. 3).

**Fig. 4:**
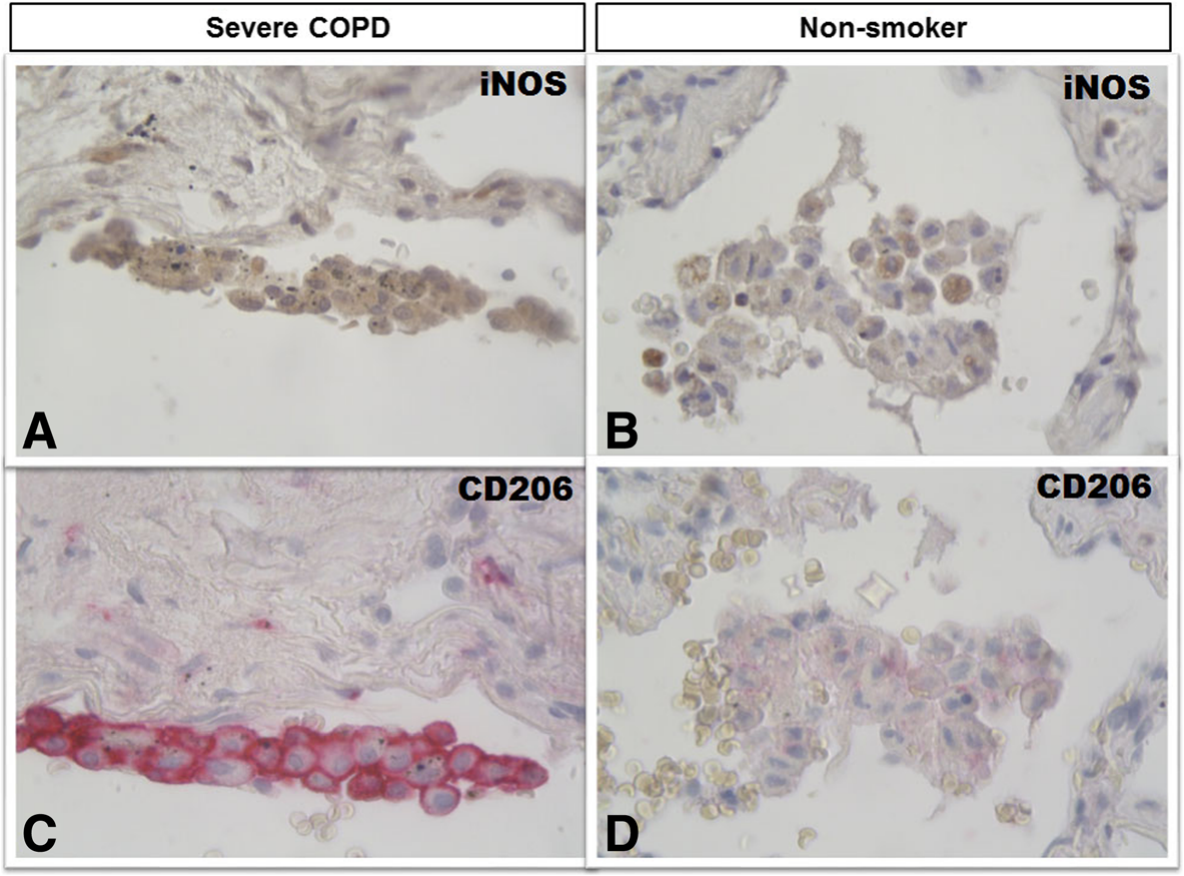
Immunohistochemistry of M1 and M2 alveolar macrophages in lung tissue. M1 (iNOS^+^) and M2 (CD206^+^) expression in clusters of alveolar macrophages in consecutive lung sections from a patient with severe COPD (panels **a** and **c**) and from a non-smoker (panels **b** and **d**). iNOS immunoreactivity appears as a brown diffuse cytoplasmic granular pattern (panel **a**), while CD206 immunoreactivity appears as a red linear pattern around the cellular membrane (panel **c**). In the smoker with severe COPD both M1 (iNOS^+^) (panel **a**) and M2 (CD206^+^) (panel **c**) immunoreactivity was present in the same cluster of alveolar macrophages. The alveolar macrophages in the non-smoking subject (panels B and D) were mostly negative for either stains. Immunostaining with anti-iNOS (in brown) and anti-CD206 (in *red*). Original magnification: X 400

**Fig. 5:**
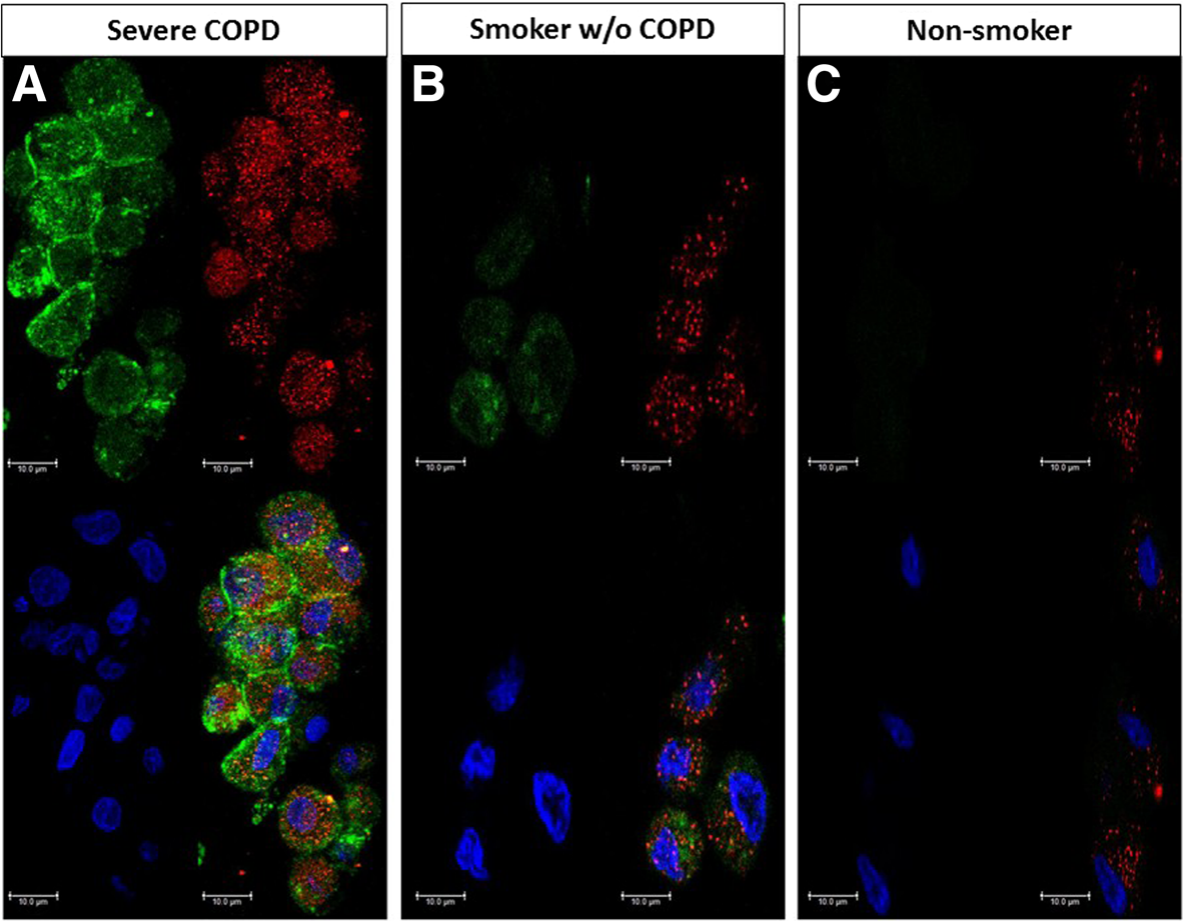
Confocal microscopy of M1 and M2 alveolar macrophages in lung tissue. iNOS (M1) and CD206 (M2) expression in clusters of alveolar macrophages in lung sections from a patient with severe COPD (panel **a**), a smoker without COPD (panel **b**) and a non-smoking subject (panel **c**). iNOS immunoreactivity appears as a red diffuse cytoplasmic granular pattern (panel **a**), while CD206 immunoreactivity appears as a green linear pattern around the cellular membrane. In the smoker with severe COPD, M1 and M2 markers were co-expressed in the same cluster of alveolar macrophages, while in the smoker without COPD only a reduced co-expression can be observed (panel **b**). The alveolar macrophages in the non-smoking subject were mostly negative for both M1 and M2 markers (panel **c**). Alveolar macrophages were stained with anti-iNOS (red) and anti-CD206 (green). Nuclei were stained with DRAQ5 (blue). Bars: 10 μm

Of interest, observing the values of both iNOS^+^ (84%) and CD206^+^ (78%) AM in severe COPD (Figs. 1 and 2), the combined value added to more than 100%, indicating that some macrophages were expressing both markers. The staining of serial sections confirmed that, in severe COPD, iNOS and CD206 immunoreactivity could be present simultaneously in the same alveolar macrophage (Fig. 4a and c). This finding was further confirmed by confocal microscopy (Fig. 5) where it could be clearly observed the co-expression of the two markers in severe COPD.

In two cases per group, we quantified the percentage of AM co-expressing both M1 and M2 markers using confocal microscopic images. We found that the percentage of AM expressing both M1 and M2 markers was 95% (range 89–100) in severe COPD, 63% (range 50–76) in moderate COPD, 35% (range 20–50) in smokers without COPD and 0 (range 0–0) in donors. Confocal analysis shows that there are also macrophages showing only M1 polarization or only M2 polarization.

Of interest, cessation of smoking significantly decreased the expression of iNOS but not of CD206 (Additional file 1: Figure S2). Indeed, when all smokers (with and without COPD) were considered together, ex-smokers had a lower percentage of iNOS^+^ AM compared to current smokers (*p =* 0.028). However, when analysed separately in the different subject groups, this difference remained statistically significant only in moderate COPD (*p <* 0.05) and in smokers without COPD (*p <* 0.01), but not in severe COPD, where the disease is fully established.

The expression of iNOS with smoking exposure and disease progression increased in parallel with the expression of TNF-α (r = 0.52; *p =* 0.002) (Additional file 1: Figure S3). Similarly, the percentage of CD206^+^ AM was paralleled by increased percentage of AM expressing IL-4 (r = 0.64; *p =* 0.004) and IL-13 (r = 0.55; *p =* 0.012) (Additional file 1: Figure S4 A and B), indicating that both M1 and M2 AM were likely active in their functions.

**Fig. 6:**
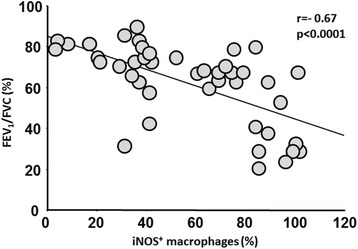
Relationship between M1 alveolar macrophages and lung function. Correlation between percentage of M1 (iNOS^+^) alveolar macrophages and FEV_1_/FVC (%) in all the subjects of the study, excluding donors who did not have lung function. Spearman rank correlation r = −0.67 and p < 0.0001

When we examined the relationship between AM polarization and lung function, we found that the percentage of AM expressing iNOS was correlated with the severity of airflow obstruction measured by the FEV_1_/FVC% (r = −0.67, *p <* 0.0001) (Fig. 6). A similar correlation was observed for CD206^+^ AM (r = −0.44, *p =* 0.003).

## Discussion

In this study we examined directly in human lung tissue the pattern of alveolar macrophage (AM) polarization, classic or alternative, and investigated how this pattern changes from the normal lung to a progressive inflammatory disease, COPD, in which the trigger is known (cigarette smoking) and the evolution of the disease can be studied functionally and pathologically. Our results showed that in healthy lungs most alveolar macrophages are neither M1 nor M2 and that, as the disease progresses, AM showed both M1 and M2 polarization that could be expressed simultaneously in the same macrophage.

We used the traditional M1 M2 classification (still widely used in the description of human macrophage polarization in the lung and other human tissues [9–14, 34–36]) in order to define, in broad terms, the response of alveolar macrophages in lung tissue to the effects of smoking and development of COPD. Studies using mice macrophages and monocyte-derived macrophages have shown that stimulation in vitro with panels of possible inflammatory and anti-inflammatory stimuli can trigger a spectrum of macrophage activation extending the M1 versus M2 polarization model [5]. The application of this knowledge to humans likely needs a disease model, perhaps like the one we are describing, in which the actual AM stimuli at the different stages of the disease can be explored.

We used iNOS, confirmed by HLADR, to define the proinflammatory M1 alveolar macrophages as suggested in the literature [10, 11, 23–27]. Both the macrophage mannose receptor, CD206, and CD163 are accepted as M2 markers in humans and have been shown, when used simultaneously in human dermis, to react similarly in the recognition of the M2 phenotype [14]. The CD206 was used for the identification of M2 in our study.

The analysis of our data allowed the direct quantification in human lung tissue of alveolar macrophages expressing either M1 or M2 or no markers of polarization, and showed how this expression changed with disease progression. In healthy lungs a high proportion of alveolar macrophages expressed no markers of either M1 or M2 activation, a finding that might have been expected in view of the tight control of the state of AM activation exerted by the alveolar epithelium, which limits unwanted inflammatory responses [34]. There has been no consensus about the state of AM polarization in health, with some reports describing alveolar macrophages as M1 while others as M2 [12, 26, 35, 36]. Recently, Desch and co-workers [37] described, in enzymatic digested lung tissue, that alveolar macrophages in healthy humans express CD206. The discrepancy with our findings might be due to the different techniques used in the two studies, and mainly to the fact that half of the subjects in Desch study were smokers or ex-smokers, and we know from the present study that smoking itself can trigger CD206 expression in alveolar macrophages.

It is believed that loss of ligands for the control of macrophages activation, like following epithelial damage during inflammation, will tip the balance towards alveolar macrophage activation into a proinflammatory M1 phenotype. Accordingly, our data show that an acute inflammatory trigger, like mechanical ventilation [33], was able to promote M1, but not M2 polarization, suggesting that acute tissue injury induces a prompt proinflammatory M1 phenotype response. With the introduction of a persistent inflammatory stimulus, cigarette smoking in our case, and the development of COPD, the proportion of proinflammatory M1 AM increases with the severity of the disease. In favour of the importance of smoking as the trigger for the proinflammatory M1 polarization is the finding that, upon smoking cessation, the percentage of AM expressing M1 decreased significantly. However, this decrease in M1 polarization is only found in smokers without COPD and smokers with mild COPD but not in smokers with severe COPD. This might suggest that the increased M1 expression in severe COPD might be due not only to cigarette smoking but also to the chronic inflammatory response characteristic of the disease.

While pathogens or tissue injury will induce the M1 phenotype, homeostatic signals from the local environment will induce macrophages to adopt phenotypes linked with tissue remodelling and repair [18], the complex M2 phenotype [38]. Whichever the signals are, AM senses tissue damage and develops a M2 phenotype to orchestrate anti-inflammatory and tissue-repair responses. In donors normal lungs, M2 polarized AM were minimally present but increased with exposure to cigarette smoking and disease severity. Of interest, the group of non-smokers who had surgery for lung tumours, showed an increase in the percentage of M2 polarization, but only if the tumour was malignant, suggesting that malignancy might influence M2 polarization [9, 39]. However, the fact that the group of smokers with severe COPD (who did not have lung cancer) had the highest percentage of M2 AM, suggests that most of the increase of M2 is secondary to smoking and COPD severity. A similar increase in M2 polarization and up-regulation of M2 related genes with worsening COPD and no lung tumour has been previously reported in bronchoalveolar lavage [35, 36], indicating that smoking and COPD severity might be the main triggers to M2 polarization, likely in response to the inflammatory reaction triggered by the M1.

Our data showed that, in smokers with COPD, the combined percentage of M1 and M2 AM added to more than 100%, indicating that some macrophages were expressing both markers. This possibility was confirmed by staining of serial sections with M1 and M2 markers and also by confocal microscopy, suggesting that not only AM might switch polarization but they could be dually polarized, expressing simultaneously both M1 and M2 markers. The expression of TNF-α or IL-4 and IL-13 as indexes of M1 and M2 activity increased along with the percentage of M1 and M2 AM respectively, indicating that both phenotypes were likely active in their functions. These data confirm in humans the fact that rather than distinct, macrophage populations with M1 and M2 signatures do not necessarily exclude each other, but often coexist as it has been shown in animal models [40] and in vitro studies [10].

This study examines for the first time the M1 and M2 AM polarization in human lung tissue. We are aware that the use of M1 and M2 as markers of polarization is only a broad approach to a much more complicated macrophage response to stimuli, as seen in animal or in vitro studies, studies that should be replicated in humans. For example, due to their location and size, human AM could be easily collected (or harvested) from human lung sections in order to study the polarization response to disease stimuli, using the newly developed single cell technologies and RNA sequencing [16, 41].

## Conclusion

In conclusion, resident alveolar macrophages in normal lungs mostly do not show either M1 or M2 polarization, however both M1 and M2 expressions increase with exposure to cigarette smoking and disease severity. The fact that alveolar macrophages can express simultaneously both markers of polarization confirms that in humans macrophage populations with M1 and M2 signatures do not necessarily exclude each other, but often coexist.

## Additional files

Additional file 1: Immunohistochemical and confocal analysis of alveolar macrophage polarization: Methods and Results. (DOCX 93 kb)

## References

[1] Wynn TA, Chawla A, Pollard JW (2013). Macrophage biology in development, homeostasis and disease. Nature.

[2] Gordon S (2007). The macrophage: past, present and future. Eur J Immunol.

[3] Martinez FO, Sica A, Mantovani A, Locati M (2008). Macrophage activation and polarization. Front Biosci.

[4] Chana KK, Fenwick PS, Nicholson AG, Barnes PJ, Donnelly LE (2014). Identification of a distinct glucocorticosteroid-insensitive pulmonary macrophage phenotype in patients with chronic obstructive pulmonary disease. J Allergy Clin Immunol.

[5] Xue J, Schmidt SV, Sander J, Draffehn A, Krebs W, Quester I, De Nardo D, Gohel TD, Emde M, Schmidleithner L, Ganesan H, Nino-Castro A, Mallmann MR, Labzin L, Theis H, Kraut M, Beyer M, Latz E, Freeman TC, Ulas T, Schultze JL (2014). Transcriptome-based network analysis reveals a spectrum model of human macrophage activation. Immunity.

[6] Mantovani A, Sica A, Allavena P, Garlanda C, Locati M (2009). Tumor-associated macrophages and the related myeloid-derived suppressor cells as a paradigm of the diversity of macrophage activation. Hum Immunol.

[7] Van Overmeire E, Laoui D, Keirsse J, Van Ginderachter JA, Sarukhan A (2014). Mechanisms driving macrophage diversity and specialization in distinct tumor microenvironments and parallelisms with other tissues. Front Immunol.

[8] Murray PJ, Allen JE, Biswas SK, Fisher EA, Gilroy DW, Goerdt S, Gordon S, Hamilton JA, Ivashkiv LB, Lawrence T, Locati M, Mantovani A, Martinez FO, Mege JL, Mosser DM, Natoli G, Saeij JP, Schultze JL, Shirey KA, Sica A, Suttles J, Udalova I, van Ginderachter JA, Vogel SN, Wynn TA (2014). Macrophage activation and polarization: nomenclature and experimental guidelines. Immunity.

[9] Conway EM, Pikor LA, Kung SH, Hamilton MJ, Lam S, Lam WL, Bennewith KL (2016). Macrophages, Inflammation, and Lung Cancer. Am J Respir Crit Care Med.

[10] Udalova IA, Mantovani A, Feldmann M (2016). Macrophage heterogeneity in the context of rheumatoid arthritis. Nat Rev Rheumatol.

[11] Ciccia F, Alessandro R, Rizzo A, Accardo-Palumbo A, Raimondo S, Raiata F, Guggino G, Giardina A, De Leo G, Sireci G, Triolo G (2014). Macrophage phenotype in the subclinical gut inflammation of patients with ankylosing spondylitis. Rheumatology.

[12] Kaku Y, Imaoka H, Morimatsu Y, Komohara Y, Ohnishi K, Oda H, Takenaka S, Matsuoka M, Kawayama T, Takeya M, Hoshino T (2014). Overexpression of CD163, CD204 and CD206 on alveolar macrophages in the lungs of patients with severe chronic obstructive pulmonary disease. PLoS One.

[13] Lee J, French B, Morgan T, French SW (2014). The liver is populated by a broad spectrum of markers for macrophages. In alcoholic hepatitis the macrophages are M1 and M2. Exp Mol Pathol.

[14] Furudate S, Fujimura T, Kambayashi Y, Kakizaki A, Aiba S (2014). Comparison of CD163+ CD206+ M2 macrophages in the lesional skin of bullous pemphigoid and pemphigus vulgaris: the possible pathogenesis of bullous pemphigoid. Dermatology.

[15] Guilliams M, De Kleer I, Henri S, Post S, Vanhoutte L, De Prijck S, Deswarte K, Malissen B, Hammad H, Lambrecht BN (2013). Alveolar macrophages develop from fetal monocytes that differentiate into long-lived cells in the first week of life via GM-CSF. J Exp Med.

[16] Ginhoux F, Schultze JL, Murray PJ, Ochando J, Biswas SK (2016). New insights into the multidimensional concept of macrophage ontogeny, activation and function. Nat Immunol.

[17] Amit I, Winter DR, Jung S (2016). The role of the local environment and epigenetics in shaping macrophage identity and their effect on tissue homeostasis. Nat Immunol.

[18] Gosselin D, Link VM, Romanoski CE, Fonseca GJ, Eichenfield DZ, Spann NJ, Stender JD, Chun HB, Garner H, Geissmann F, Glass CK (2014). Environment drives selection and function of enhancers controlling tissue-specific macrophage identities. Cell.

[19] Lavin Y, Winter D, Blecher-Gonen R, David E, Keren-Shaul H, Merad M, Jung S, Amit I (2014). Tissue-resident macrophage enhancer landscapes are shaped by the local microenvironment. Cell.

[20] Bazzan E, Saetta M, Turato G, Borroni EM, Cancellieri C, Baraldo S, Savino B, Calabrese F, Ballarin A, Balestro E, Mantovani A, Cosio MG, Bonecchi R, Locati M (2013). Expression of the atypical chemokine receptor D6 in human alveolar macrophages in COPD. Chest.

[21] Klug F, Prakash H, Huber PE, Seibel T, Bender N, Halama N, Pfirschke C, Voss RH, Timke C, Umansky L, Klapproth K, Schäkel K, Garbi N, Jäger D, Weitz J, Schmitz-Winnenthal H, Hämmerling GJ, Beckhove P (2013). Low-dose irradiation programs macrophage differentiation to an iNOS^+^/M1 phenotype that orchestrates effective T cell immunotherapy. Cancer Cell.

[22] Mills CD (2001). Macrophage arginine metabolism to ornithine/urea or nitric oxide/citrulline: a life or death issue. Crit Rev Immunol.

[23] Stöger JL, Gijbels MJ, van der Velden S, Manca M, van der Loos CM, Biessen EA, Daemen MJ, Lutgens E, de Winther MP (2012). Distribution of macrophage polarization markers in human atherosclerosis. Atherosclerosis.

[24] Quatromoni JG, Eruslanov E (2012). Tumor-associated macrophages: function, phenotype, and link to prognosis in human lung cancer. Am J Transl Res.

[25] Ohri CM, Shikotra A, Green RH, Waller DA, Bradding P (2009). Macrophages within NSCLC tumour islets are predominantly of a cytotoxic M1 phenotype associated with extended survival. Eur Respir J.

[26] Hodge S, Matthews G, Mukaro V, Ahern J, Shivam A, Hodge G, Holmes M, Jersmann H, Reynolds PN (2011). Cigarette smoke-induced changes to alveolar macrophage phenotype and function are improved by treatment with procysteine. Am J Respir Cell Mol Biol.

[27] Ma J, Liu L, Che G, Yu N, Dai F, You Z (2010). The M1 form of tumor-associated macrophages in non-small cell lung cancer is positively associated with survival time. BMC Cancer.

[28] Deng W, Chen W, Zhang Z, Huang S, Kong W, Sun Y, Tang X, Yao G, Feng X, Chen W, Sun L (2015). Mesenchymal stem cells promote CD206 expression and phagocytic activity of macrophages through IL-6 in systemic lupus erythematosus. Clin Immunol.

[29] Kambara K, Ohashi W, Tomita K, Takashina M, Fujisaka S, Hayashi R, Mori H, Tobe K, Hattori Y (2015). In vivo depletion of CD206+ M2 macrophages exaggerates lung injury in endotoxemic mice. Am J Pathol.

[30] Hirata Y, Tabata M, Kurobe H, Motoki T, Akaike M, Nishio C, Higashida M, Mikasa H, Nakaya Y, Takanashi S, Igarashi T, Kitagawa T, Sata M (2011). Coronary atherosclerosis is associated with macrophage polarization in epicardial adipose tissue. J Am Coll Cardiol.

[31] Veremeyko T, Siddiqui S, Sotnikov I, Yung A, Ponomarev ED (2013). IL-4/IL-13-dependent and independent expression of miR-124 and its contribution to M2 phenotype of monocytic cells in normal conditions and during allergic inflammation. PLoS One.

[32] Stein M, Keshav S, Harris N, Gordon S (1992). Interleukin 4 potently enhances murine macrophage mannose receptor activity: a marker of alternative immunologic macrophage activation. J Exp Med.

[33] D’Angelo E, Pecchiari M, Saetta M, Balestro E, Milic-Emili J (2004). Dependence of lung injury on inflation rate during low-volume ventilation in normal open- chest rabbits. J Appl Physiol.

[34] Hussell T, Bell TJ (2014). Alveolar macrophages: plasticity in a tissue-specific context. Nat Rev Immunol.

[35] Kunz LI, Lapperre TS, Snoeck-Stroband JB, Budulac SE, Timens W, van Wijngaarden S, Schrumpf JA, Rabe KF, Postma DS, Sterk PJ, Hiemstra PS (2011). Smoking status and anti-inflammatory macrophages in bronchoalveolar lavage and induced sputum in COPD. Respir Res.

[36] Shaykhiev R, Krause A, Salit J, Strulovici-Barel Y, Harvey BG, O’Connor TP, Crystal RG (2009). Smoking-dependent reprogramming of alveolar macrophage polarization: implication for pathogenesis of chronic obstructive pulmonary disease. J Immunol.

[37] Desch AN, Gibbings SL, Goyal R, Kolde R, Bednarek J, Bruno T, Slansky JE, Jacobelli J, Mason R, Ito Y, Messier E, Randolph GJ, Prabagar M, Atif SM, Segura E, Xavier RJ, Bratton DL, Janssen WJ, Henson PM, Jakubzick CV (2016). Flow Cytometric Analysis of Mononuclear Phagocytes in Nondiseased Human Lung and Lung-Draining Lymph Nodes. Am J Respir Crit Care Med.

[38] Mantovani A, Sica A, Sozzani S, Allavena P, Vecchi A, Locati M (2004). The chemokine system in diverse forms of macrophage activation and polarization. Trends Immunol.

[39] Biswas SK, Mantovani A (2010). Macrophage plasticity and interaction with lymphocyte subsets: cancer as a paradigm. Nat Immunol.

[40] Patil V, Zhao Y, Shah S, Fox BA, Rommereim LM, Bzik DJ, Yap GS (2014). Co-existence of classical and alternative activation programs in macrophages responding to Toxoplasma gondii. Int J Parasitol.

[41] Jaitin DA, Kenigsberg E, Keren-Shaul H, Elefant N, Paul F, Zaretsky I, Mildner A, Cohen N, Jung S, Tanay A, Amit I (2014). Massively parallel single-cell RNA-seq for marker-free decomposition of tissues into cell types. Science.

