# Supplementary material for: Dual polarization of human alveolar macrophages progressively increases with smoking and COPD severity
Source: Respir Res. 2017 Feb 23;18:40. doi: 10.1186/s12931-017-0522-0 (PMC5324331; doi:10.1186/s12931-017-0522-0)
Supplement: Additional file 1: — Immunohistochemical and confocal analysis of alveolar macrophage polarization: Methods and Results. (DOCX 93 kb) [file 12931_2017_522_MOESM1_ESM.docx]

Dual polarization of human alveolar macrophages progressively increases with smoking and COPD severity

Erica Bazzan^1^, Graziella Turato^1^, Mariaenrica Tinè^1^, Claudia M. Radu^2^, Elisabetta Balestro^1^, Chiara Rigobello^1^, Davide Biondini^1^, Marco Schiavon^1^, Francesca Lunardi^1^, Simonetta Baraldo^1^, Federico Rea^1^, Paolo Simioni^2^, Fiorella Calabrese^1^, Marina Saetta^1^ and Manuel G. Cosio^1,3^

1 Department of Cardiac, Thoracic, and Vascular Sciences, University of Padova, Italy;

2 Department of Medicine, University of Padova, Italy

3 Respiratory Division, Meakins-Christie Laboratories, McGill University, Montreal, Canada

**Additional file 1: immunohistochemical and confocal analysis of alveolar macrophage polarization: methods and results.**

**Materials and Methods**

***Tissue sample analysis and immunohistochemistry.***

For the present study, lung tissue from 53 subjects undergoing surgery for appropriate clinical indications were collected and categorized into the following five groups: smokers with severe COPD (n=11); smokers with moderate COPD (n=12); smokers with normal lung function (smokers w/o COPD n=13); non-smoking subjects with normal lung function (non-smokers; n=11) and donors (n=6). All patients with severe COPD underwent lung volume reduction surgery (LVRS) and did not have lung cancer. Samples from moderate COPD, smokers w/o COPD and non-smokers were obtained from subjects undergoing lobectomy for suspected lung cancer. Six non-smokers had cancer while 5 had a benign tumour. Donors lungs were non implanted lungs. All patients with severe COPD were treated with inhaled anticholinergics and/or β2 agonists/corticosteroids.

In specimens obtained at surgery (LVRS or lobectomy), randomly selected tissue blocks were taken from the subpleural parenchyma, as far as possible from the area with tumour in patients who underwent lung resection for nodules. Samples were fixed in 10% phosphate-buffered formalin (pH 7.2) for 24 hours and embedded in paraffin wax (S1). 5 μm-thick sections were cut and processed for immunohistochemical analysis of iNOS, HLA-DR and CD206 (S2-S15). Additionally in a subgroup of patients (equally represented among the different groups examined) the expression of TNF-α (n=37), IL-4 (n=21) and IL-13 (n=22) in AM was investigated as indexes of M1 (TNF-α) and M2 (IL-4 and IL-13) activity (S16, S17). Details are reported in Table S1.

TABLE S1:

| Antibody | Dilution | Company and Catalogue number | Antigen retrieval method | Antigen detection |
| --- | --- | --- | --- | --- |
| Polyclonal rabbit anti-iNOS | 1:1000 (overnight) | Thermo Fisher Scientific, UK | Microwave in citrate buffer pH 6 | Envision detection system, peroxidase and diaminobenzidine (DAB) |
| Monoclonal mouse anti-CD206 | 1:50 | R&D Systems, Milan, Italy | Microwave in citrate buffer pH 6 | Anti-mouse immunoglobulins coniugated with alkaline phosphatase and liquid permanent red |
| Monoclonal mouse anti-HLA-DR | 1:50 | DAKO, UK | Microwave in citrate buffer pH 6 | Anti-mouse immunoglobulins coniugated with alkaline phosphatase and liquid permanent red |
| Monoclonal mouse anti-TNFα | 1:200 | Sigma-Aldrich, St. Louis, MO | Microwave in citrate buffer pH 6 | Envision detection system, peroxidase and diaminobenzidine (DAB) |
| Polyclonal rabbit anti-IL-4 | 1:300 | PeproTech, UK | Microwave in citrate buffer pH 6 | Envision detection system, peroxidase and diaminobenzidine (DAB) |
| Polyclonal rabbit anti-IL-13 | 1:300 | PeproTech, UK | Microwave in citrate buffer pH 6 | Envision detection system, peroxidase and diaminobenzidine (DAB) |

Negative controls for nonspecific binding were processed either omitting the primary antibody or using isotype IgG and revealed no signal.

To quantify iNOS, CD206, HLA-DR, TNFα, IL-4 and IL-13 expression in alveolar macrophages, at least 20 non consecutive high-power fields (HPF) and at least 100 macrophages inside the alveolar spaces were evaluated for each patient; results were expressed as percentage of iNOS, CD206, HLA-DR, TNFα, IL-4 and IL-13 positive macrophages over the total number of macrophages examined (S1). Alveolar macrophages were defined as mononuclear cells with a well-represented cytoplasm, present in the alveolar spaces.

***Confocal microscopy***

Confocal microscopy was used to investigate the possible coexpression of iNOS and CD206. After dewaxing and rehydrating through serial alcohols, sections were treated with 0.5% Triton X-100 in phosphate buffered saline (PBS, pH 7.4) for 15 minutes at room temperature (RT) to permeabilize the cells. Subsequently, after washing with PBS sections were treated with 0.05 M NH4Cl for 15 minutes at room temperature. Afterwards, tissue samples were stained both with 1:40 diluted rabbit anti-human-iNOS (Thermo Fisher Scientific, UK) and 1:60 diluted mouse anti-human-CD206 (R&D Systems, Milan, Italy) incubated over night at 4°C. Following two rinsing procedures with PBS, samples were incubated for one hour at 37°C with 1:300 diluted tetramethylrhodamine-isothiocyanate (TRITC) conjugated antibody (Life Technologies, Carlsbad, USA) goat anti-rabbit-IgG Alexa Fluor® 594. Subsequently, after washing with PBS the samples were incubated for one hour with 1:200 diluted fluorescein isothiocyanate (FITC) conjugated antibody goat anti-mouse IgG (Sigma-Aldrich). Secondary antibodies were also used in the absence of primary antibodies in order to assess non-specific binding. The primary and secondary antibodies were diluted in PBS containing 0.5% bovine serum albumin (Sigma-Aldrich). For confocal analysis, nuclei were stained using DRAQ5TM (Abcam, Cambridge, UK) for 30 minutes at 37°C. Afterwards, after two rinsing procedures with PBS, slides were mounted with mowiol antifade medium (Sigma-Aldrich). Samples were analysed by a confocal microscope Leica TCS SP8 (Leica Microsystems, Wetzlar, Germany) with a z-stack around 1 μm and using a 63x/1.4 oil immersion lens (image size 1024x1024 pixels) and images acquired using a DFC365FX camera (Leica, Microsystems). The expressions of the two markers were performed with Leica Application Suite (LAS-AF) 3.1.1 software.

***Statistical analysis***

All cases were coded and the measurements were made without knowledge of clinical data to avoid observer bias. Differences between groups were evaluated with either ANOVA (for the clinical data) or the Kruskal-Wallis test (for the morphological data). When significant differences among groups were observed a paired comparison between two groups was performed using Student t tests (for the clinical data) or Mann-Whitney U tests (for the morphological data). Correlation coefficients were calculated using the nonparametric Spearman rank method. P values of 0.05 or less were considered to indicate statistical significance. In a subgroup of patients, the same measurements were performed again by the same observer to assess the intraobserver reproducibility, and by a second observer to assess the interobserver reproducibility. The intraobserver correlation coefficiecient for iNOS and CD206 was 0.90 and 0.94 respectively, while the interobserver correlation coefficient was 0.88 and 0.90.

Results

When non-smokers were divided on the presence or absence of malignant tumour, the percentage of CD206^+^ AM was significantly increased in non-smokers with malignant tumour compared to those with benign tumour. However, the percentage of iNOS^+^ AM was similar (Figure S1).

Figure S1: Percentage of iNOS^+^ and CD206^+^ AM in non-smokers with malignant tumour and in those with benign tumour. Bottom and top of each box plot, 25^th^ and 75^th^ percentiles; solid line, median; brackets, 10^th^ and 90^th^ percentiles.

Cessation of smoking significantly decreased the expression of iNOS, as shown by the lower percentage of iNOS^+^ AM in ex-smokers compared to current smokers (Figure S2). However, when analysed by groups, this difference remained statistically different in moderate COPD (p<0.05) and smokers without COPD (p<0.01), but not in severe COPD where the disease was fully established. Smoking cessation did not influence CD206 expression.

Figure S2: Percentage of iNOS^+^ and CD206+ AM in current and ex-smokers. Bottom and top of each box plot, 25^th^ and 75^th^ percentiles; solid line, median; brackets, 10^th^ and 90^th^ percentiles.

In our study, classical activated M1 alveolar macrophages were defined by the use of iNOS and confirmed by HLA-DR. As for iNOS, the percentage of HLA-DR^+^ AM increased with smoking and disease severity from 0(0-6) in donors to 57(46-88) in severe COPD. The percentage of iNOS^+^ AM correlated with that of TNFα^+^ AM (Figure S3). Similarly, the amount of CD206^+^ AM correlated with the percentage of IL-4^+^ and IL-13^+^ AM (Figure S4). The percentage of TNFα^+^ as well as that of IL-4^+^ and IL-13^+^  AM was lower in donors and non-smokers compared to smokers with and w/o COPD (p<0.05 for all comparisons).

Figure S3: Relationship between the percentage of iNOS^+^ AM and the percentage of TNFα^+^ AM in the subjects in whom TNFα was quantified. Spearman rank correlation r=0.52 and p= 0.002. Smokers with COPD; Smokers w/o COPD; Non-smokers; Donors.

**B**

**A**

Figure S4: Relationship between the percentage of CD206^+^ AM and the percentage of IL-4^+^ (A) and IL-13^+^ (B) AM in the subjects in whom IL-4 and IL-13 were quantified. Spearman rank correlation r=0.64 and p= 0.004 and r=0.55 and p= 0.012 respectively. Smokers with COPD; Smokers w/o COPD; Non-smokers; Donors.

REFERENCES

S1. Bazzan E, Saetta M, Turato G, Borroni EM, Cancellieri C, Baraldo S, Savino B, Calabrese F, Ballarin A, Balestro E, Mantovani A, Cosio MG, Bonecchi R, Locati M. Expression of the atypical chemokine receptor D6 in human alveolar macrophages in COPD. Chest. 2013;143:98-106.

S2. Klug F, Prakash H, Huber PE, Seibel T, Bender N, Halama N, Pfirschke C, Voss RH, Timke C, Umansky L, Klapproth K, Schäkel K, Garbi N, Jäger D, Weitz J, Schmitz-Winnenthal H, Hämmerling GJ, Beckhove P. Low-dose irradiation programs macrophage differentiation to an iNOS⁺/M1 phenotype that orchestrates effective T cell immunotherapy. Cancer Cell. 2013;24:589-602.

S3. Mills CD. Macrophage arginine metabolism to ornithine/urea or nitric oxide/citrulline: a life or death issue. Crit Rev Immunol. 2001;21:399-425.

S4. Ciccia F, Alessandro R, Rizzo A, Accardo-Palumbo A, Raimondo S, Raiata F, Guggino G, Giardina A, De Leo G, Sireci G, Triolo G. Macrophage phenotype in the subclinical gut inflammation of patients with ankylosing spondylitis. Rheumatology. 2014;53:104-13.

S5. Stöger JL, Gijbels MJ, van der Velden S, Manca M, van der Loos CM, Biessen EA, Daemen MJ, Lutgens E, de Winther MP. Distribution of macrophage polarization markers in human atherosclerosis. Atherosclerosis. 2012;225:461-8

S6. Quatromoni JG, Eruslanov E. Tumor-associated macrophages: function, phenotype, and link to prognosis in human lung cancer. Am J Transl Res. 2012;4:376-89.

S7. Ohri CM, Shikotra A, Green RH, Waller DA, Bradding P. Macrophages within NSCLC tumour islets are predominantly of a cytotoxic M1 phenotype associated with extended survival. Eur Respir J. 2009;33:118-26.

S8. Hodge S, Matthews G, Mukaro V, Ahern J, Shivam A, Hodge G, Holmes M, Jersmann H, Reynolds PN. Cigarette smoke-induced changes to alveolar macrophage phenotype and function are improved by treatment with procysteine. Am J Respir Cell Mol Biol. 2011;44:673-81.

S9. Ma J, Liu L, Che G, Yu N, Dai F, You Z. The M1 form of tumor-associated macrophages in non-small cell lung cancer is positively associated with survival time. BMC Cancer. 2010;10:112.

S10. Deng W, Chen W, Zhang Z, Huang S, Kong W, Sun Y, Tang X, Yao G, Feng X, Chen W, Sun L. Mesenchymal stem cells promote CD206 expression and phagocytic activity of macrophages through IL-6 in systemic lupus erythematosus. Clin Immunol. 2015;161:209-16.

S11. Kambara K, Ohashi W, Tomita K, Takashina M, Fujisaka S, Hayashi R, Mori H, Tobe K, Hattori Y. In vivo depletion of CD206+ M2 macrophages exaggerates lung injury in endotoxemic mice. Am J Pathol. 2015;185:162-71.

S12. Hirata Y, Tabata M, Kurobe H, Motoki T, Akaike M, Nishio C, Higashida M, Mikasa H, Nakaya Y, Takanashi S, Igarashi T, Kitagawa T, Sata M. Coronary atherosclerosis is associated with macrophage polarization in epicardial adipose tissue. J Am Coll Cardiol. 2011;58:248-55.

S13. Kaku Y, Imaoka H, Morimatsu Y, Komohara Y, Ohnishi K, Oda H, Takenaka S, Matsuoka M, Kawayama T, Takeya M, Hoshino T. Overexpression of CD163, CD204 and CD206 on alveolar macrophages in the lungs of patients with severe chronic obstructive pulmonary disease. PLoS One. 2014;9:e87400.

S14. Lee J, French B, Morgan T, French SW. The liver is populated by a broad spectrum of markers for macrophages. In alcoholic hepatitis the macrophages are M1 and M2. Exp Mol Pathol. 2014;96:118-25.

S15. Furudate S, Fujimura T, Kambayashi Y, Kakizaki A, Aiba S. Comparison of CD163+ CD206+ M2 macrophages in the lesional skin of bullous pemphigoid and pemphigus vulgaris: the possible pathogenesis of bullous pemphigoid. Dermatology. 2014;229:369-78.

S16. Veremeyko T, Siddiqui S, Sotnikov I, Yung A, Ponomarev ED. IL-4/IL-13-dependent and independent expression of miR-124 and its contribution to M2 phenotype of monocytic cells in normal conditions and during allergic inflammation. PLoS One. 2013;8:e81774.

S17. Stein M, Keshav S, Harris N, Gordon S. Interleukin 4 potently enhances murine macrophage mannose receptor activity: a marker of alternative immunologic macrophage activation. J Exp Med. 1992;176:287-292.
